# Electrochemical study of 4-chloroaniline in a water/acetonitrile mixture. A new method for the synthesis of 4-chloro-2-(phenylsulfonyl)aniline and *N*-(4-chlorophenyl)benzenesulfonamide†

**DOI:** 10.1039/d0ra05680d

**Published:** 2020-08-26

**Authors:** Niloofar Mohamadighader, Mahnaz Saraei, Davood Nematollahi, Hamed Goljani

**Affiliations:** Department of Chemistry, Payame Noor University Tehran Iran; Faculty of Chemistry, Bu-Ali Sina University Hamedan 65174 Iran nemat@basu.ac.ir

## Abstract

The electrochemical oxidation of 4-chloroaniline as a model compound in a water/acetonitrile mixture was investigated by cyclic voltammetry and differential pulse voltammetry. It was established that one-electron oxidation of 4-chloroaniline followed by disproportionation reaction affords unstable (4-iminocyclohexa-2,5-dien-1-ylidene)chloronium. In water/acetonitrile mixtures and in the absence of nucleophiles, the most likely reaction on produced chloronium is hydrolysis and *p*-quinoneimine formation. The electrochemical oxidation of 4-chloroaniline in the presence of arylsulfinic acids was also investigated in a water/acetonitrile mixture at a glassy carbon electrode. It was established that under these conditions, the anodically generated chloronium reacts with benzenesulfinic acid to produce the corresponding diaryl sulfone and *N*-phenylbenzenesulfonamide derivatives. In addition, Gaussian 09W was applied for prediction of the possible product by the calculation of natural charge, LUMO orbital energies and thermodynamic stability of intermediates and products.

## Abstract 2

Electrochemical oxidation pathway of *p*-chloroaniline (PCA) in the presence of benzenesulfinic acid (BSA).

## Graphical abstract

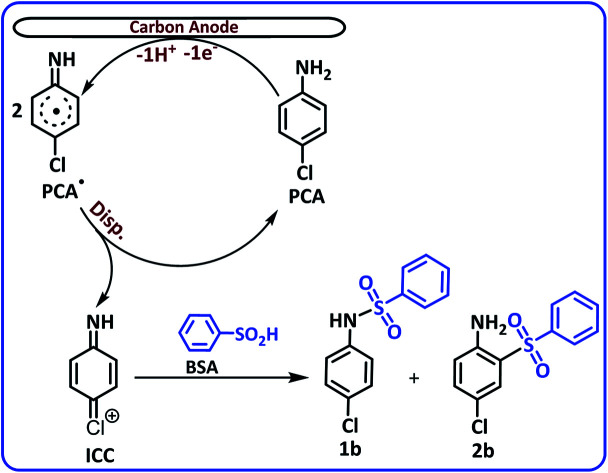

## Introduction

Haloaniline derivatives are important building blocks in the synthesis of polymers, dyes, agricultural chemicals and pharmaceuticals.^1^ Among them, 4-chloroaniline or *p*-chloroaniline, PCA, is utilized as an intermediate in the synthesis of a number of pharmaceuticals, herbicides and insecticides (*e.g.*, monuron, diflubenzuron), pigments, azo dyes and cosmetic products. It is also used as a precursor in the manufacture of pesticides, such as chlorphthalim, anilofos, monolinuron and pyraclostrobin.^2–5^ Despite the large number of studies focused on the synthetic aspects of PCA, a literature survey shows that the electrochemical information on oxidation behavior of PCA in aqueous solutions is very limited. The first published paper is related to the study by Adams *et al.*^6^ They have shown that in acidic solutions, the first step in the oxidation of PCA is the formation of PCA radical cation and the second step is dimerization of the radicals. Reddy and coworker studied oxidation of PCA in aqueous solutions at a platinum electrode and suggested that the electrochemical oxidation of PCA in alkaline media, leads to the formation of 4,4′-dichloroazobenzene.^7^ The electrodimerization mechanism of PCA was studied by Amatore and coworkers in unbuffered DMF medium. They used conventional and fast voltammetry to show that the primary radical cation intermediates, formed by the one electron oxidation of PCA.^8^ Kadar *et al.* studied electrochemical oxidation of 4-chloroaniline in acetonitrile and stated that “*the substituent in the para position is not only eliminated at the electrochemically initiated dimerization step, but it was oxidized to chlorine, which substitutes the free ortho position of the starting chloroaniline*”.^9^ In addition, other researchers have studied the oxidation of this compound and published valuable information.^10–12^ Since, a careful choice of electrochemical parameters are necessary for a successful electrochemical synthesis, in the first step, we studied the electrochemical behavior of PCA in the water/acetonitrile mixture (solvent of synthesis), so that we could use the data for synthetic purposes.

**Fig. 1:**
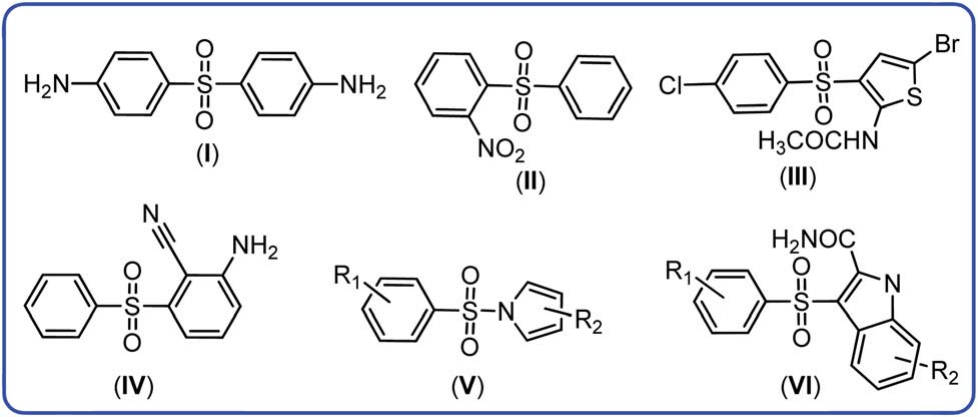
The structures of dapsone (I), 2-nitrophenyl phenyl sulfone (II), *N*-(5-bromo-3-((4-chlorophenyl)sulfonyl)thiophen-2-yl)acetamide (III), 2-amino-6-arylsulfonylbenzonitrile (IV), pyrrylarylsulfone (V) and indolylaryl sulfones (VI).

Since diaryl sulfone and *N*-phenylbenzenesulfonamide derivatives have been synthesized in this work, in this section, we will point out the importance of these compounds as well as a brief discussion covering other synthetic strategies for the synthesis of these compounds. Diaryl sulfones are useful intermediates due to their interesting biological^13–17^ and chemical^18–20^ properties. Diaryl sulfone derivatives are either an antibiotic like dapsone for the treatment of leprosy^13^ or have anti-HIV-1 activity like 2-nitrophenyl phenyl sulfone,^14^*N*-(5-bromo-3-((4-chlorophenyl)sulfonyl)thiophen-2-yl)acetamide,^15^ 2-amino-6-arylsulfonylbenzonitrile,^16^ pyrrylarylsulfone^17^ and indolylaryl sulfones^14^ (Fig. 1).

The classical method for the preparation of diaryl sulfone involves the reaction between amines,^21^ boronic acids,^22–24^ aryl halides,^25^ phenyliodonium salts,^26^ and arenediazonium tetrafluoroborates and sodium sulfinates^27^ or sulfonyl azides^28^ as sulfonyl donors. In addition, the oxidation of sulphides and sulfoxides are efficient methods for the synthesis of diaryl sulfone.^29,30^ In addition, diaryl sulfones were synthesized by Ni-catalysed photoredox sulfinylation of halides,^31^ cross coupling of aryl iodide with sulfinate salts in the presence of photoredox/Ni catalyst,^32^ Cu-catalyzed direct sulfonylation of indolines,^33^ silver-promoted decarboxylative sulfonylation of aromatic carboxylic acids with sodium sulfinates,^34^ arenes and 3CdSO_4_·*x*H_2_O in the presence of P_2_O_5_*via* mechanochemistry^35^ and electrochemical methods.^36–38^ All of these methods are efficient in synthesis of diaryl sulfones. They all have strengths, capabilities and weaknesses. The aim of this study is to provide a thorough understanding of the oxidation pathways of 4-chloroaniline as a model compound and establishes a new strategy for the synthesis of new diaryl sulfone and *N*-phenylbenzenesulfonamide derivatives along with previous methods.

## Results and discussion

**Fig. 2:**
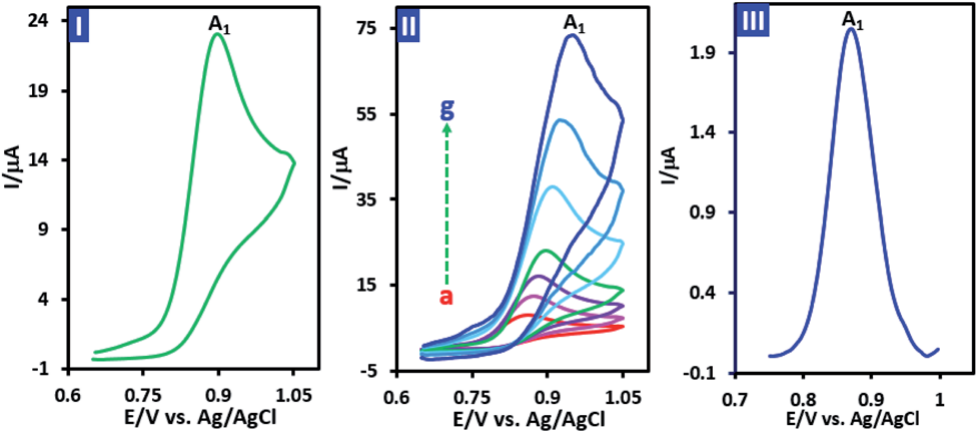
Part I: cyclic voltammogram of 1.0 mM PCA at scan rate of 100 mV s^−1^. Part II: cyclic voltammograms of PCA (1.0 mM) at different scan rates. Scan rates from a to g are: 10, 25, 50, 100, 250, 500 and 1000 mV s^−1^. Part III: differential pulse voltammogram of PCA (0.1 mM). Solvent: water (phosphate buffer, pH, 2.0, *c* = 0.2 M)/acetonitrile (50/50 v/v) mixture at GC electrode. Step potential: 5 mV, modulation amplitude: 25 mV, modulation time: 0.05 s, at room temperature.

The cyclic voltamogram of *p*-chloroaniline (PCA) (1.0 mM) in water (phosphate buffer, pH, 2.0, *c* = 0.2 M)/acetonitrile (50/50 v/v) mixture is shown in Fig. 2, part I. The voltammogram consists of an irreversible anodic peak (A_1_) which shows the instability of oxidized PCA. The irreversible nature of oxidation of PCA, observed at all studied scan rates (10 to 1000 mV s^−1^) (Fig. 2, part II), clearly confirms the limited stability of oxidized PCA. In other words, due to the higher reactivity of oxidized PCA and its conversion to other compounds, there is no sign of the reduction peak. In order to calculate the number of electrons exchanged in the oxidation of PCA in a short time scale, the half-peak width (*W*_1/2_) of differential pulse voltammogram of PCA was used (eqn (1)). The differential pulse voltammogram of PCA is shown in Fig. 2, part III. The measured half-peak width (*W*_1/2_) is 75 mV. Based on this, the number of exchanged electrons is calculated to be 1.2.

1 *W*_1/2_ = 3.52*RT*/*nF*

**Fig. 3:**
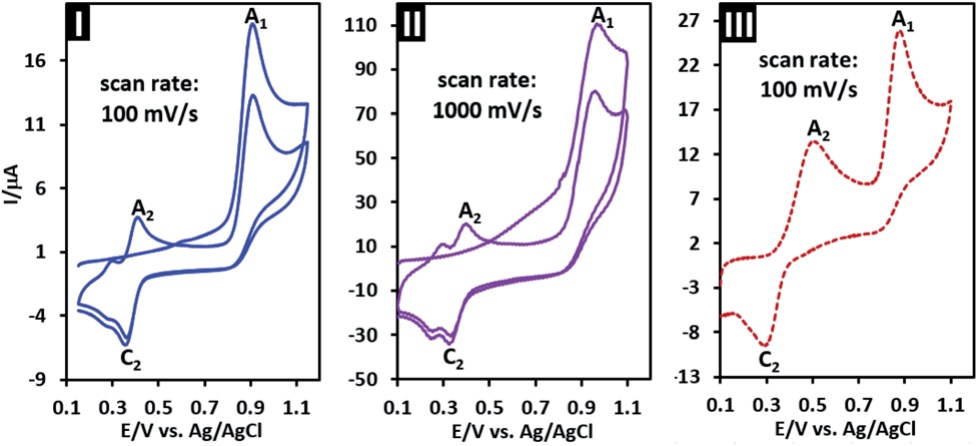
Part I, cyclic voltammograms (first and second cycles) of 1.0 mM PCA at scan rate: 100 mV s^−1^. Part II, similar to part I at scan rate: 1000 mV s^−1^. Part III, cyclic voltammogram (second cycle) of 1.0 mM PCA in the presence of 0.25 mM PAP at scan rate: 100 mV s^−1^. Solvent: water (phosphate buffer, pH, 2.0, *c* = 0.2 M)/acetonitrile (50/50 v/v) at GC electrode at room temperature.

The first and second cycles of the cyclic voltammogram of PCA at two potential scan rates are shown in Fig. 3 parts I and II. In the first cycle, CVs show the mentioned anodic peak A_1_ and independent cathodic peak C_2_. In the second cycle, a new anodic peak (A_2_) which is the counterpart of peak C_2_ appears. This behavior is a clear example of a typical ECE mechanism.^39^Fig. 3 part III shows the second cycle of the cyclic voltammogram of PCA in the similar conditions with that of part I, but with the difference that *p*-aminophenol (PAP) (0.25 mM) is added to the solution. As can be seen, in the presence of PAP, *I*_pA2_ increases, which confirms that peak A_2_ is related to the oxidation of PAP.

**Scheme 1:**
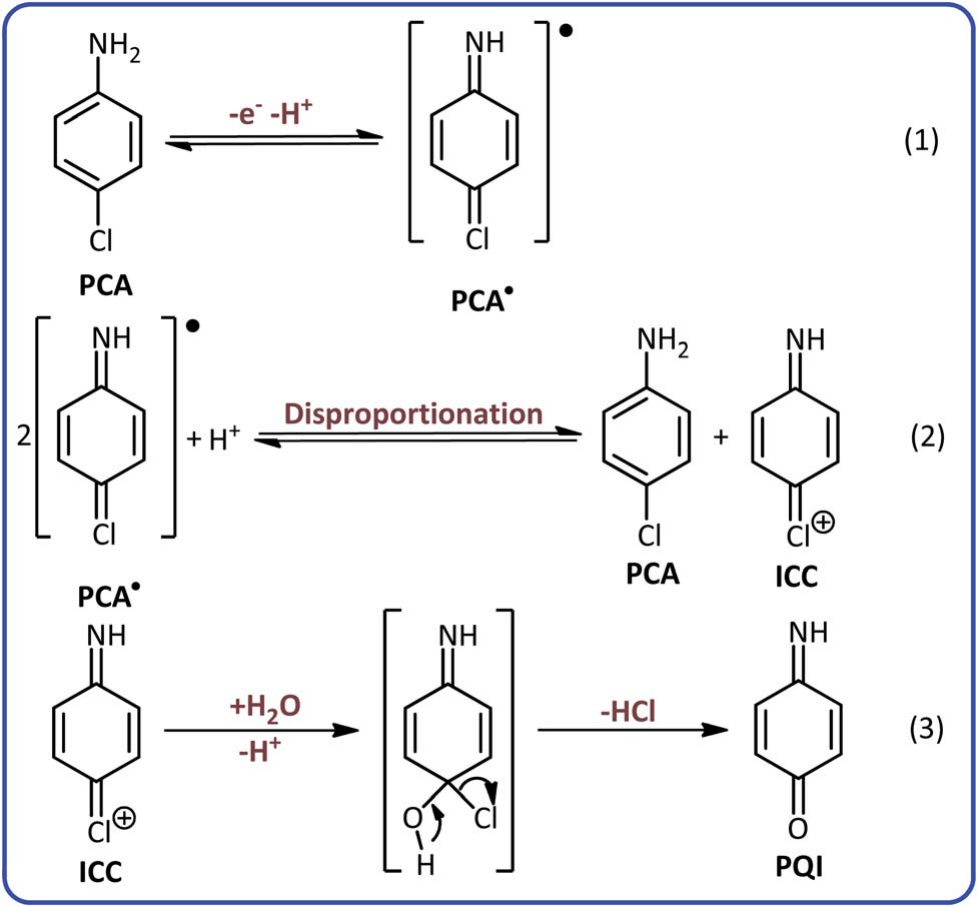
Electrochemical oxidation pathway of PCA in aqueous solutions.

According to these results and previous data,^40,41^ it can be concluded that the peaks A_2_ and C_2_ are related to the oxidation of *p*-aminophenol (PAP) to *p*-quinoneimine (PQI) and reduction of PQI to PAP, respectively. Considering all the available information, the following mechanism can be considered for electrochemical oxidation of PCA in aqueous solutions (Scheme 1).

According to Scheme 1, the PCA is first converted to the corresponding radical (PCA˙) after the loss of an electron and a proton. The disproportionation reaction in the next step, converts PCA˙ into (4-iminocyclohexa-2,5-dien-1-ylidene)chloronium (ICC). ICC is an unstable compound that in the time scale of our voltammetric experiments and in acidic solution, is hydrolyzed to *p*-quinoneimine (PQI).

**Fig. 4:**
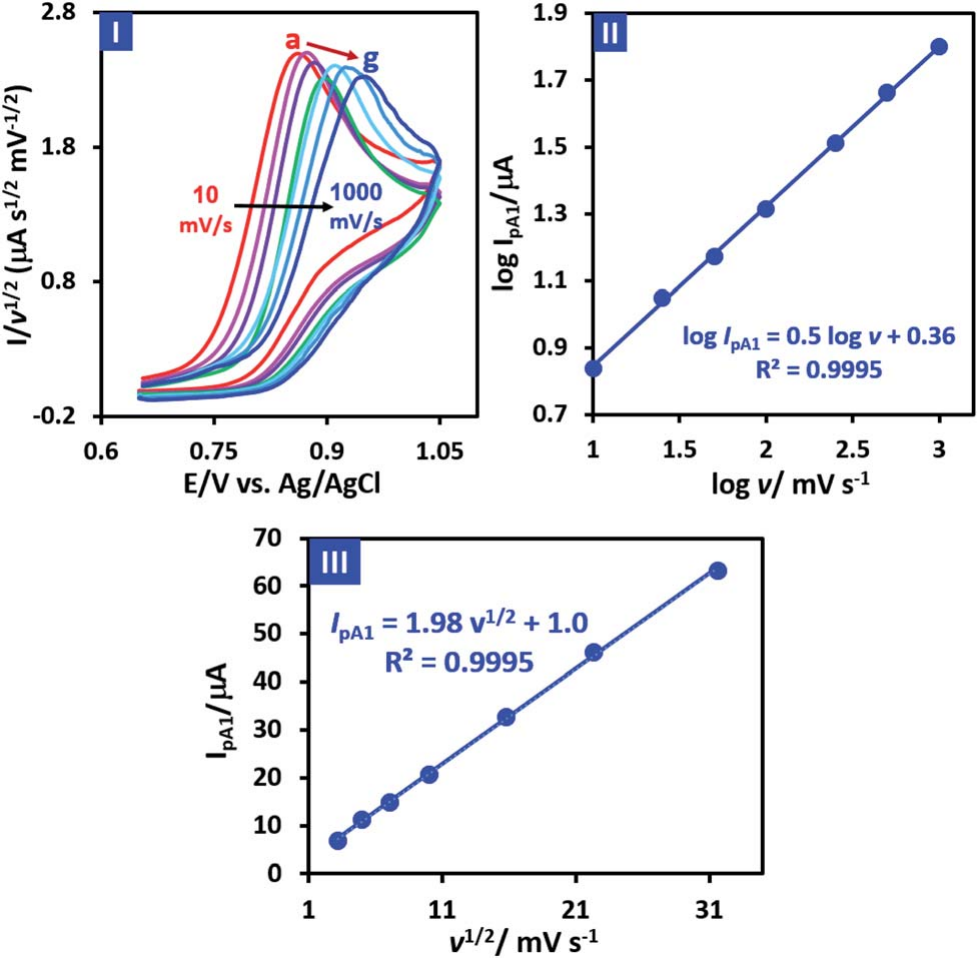
Part I: normalized cyclic voltammograms of 1.0 mM PCA at different scan rates (10, 25, 50, 100, 250, 500 and 1000 mV s^−1^). Part II: plot of log *I*_pA1_*vs.* log *v*. Part III: plot of *I*_pA1_*vs. v*^1/2^. The data are taken from cyclic voltammograms shown in Fig. 2 part II.

In order to obtain more information about the electrochemical oxidation of PCA, its diffusion/adsorption characteristics were examined. For this purpose, the normalized cyclic voltammograms of PCA is shown in Fig. 4, part I. It should be noted that the data used in Fig. 4 (parts I and II), are taken from cyclic voltammograms shown in Fig. 2 part II. It is also necessary to mention that the normalization was carried out by dividing the current on the square root of the potential scan rate (*I*/*v*^1/2^).

Two important points can be deduced from Fig. 4 part I. First, the normalized peak current (*I*_pA1_/*v*^1/2^) for different scan rates is almost the same, which is evidence of the diffusion nature of PCA oxidation under experimental conditions.^39^ The second point is that the peak potential (*E*_pA1_) shifts towards positive potentials as scan rate increases. This feature is a characteristic property of an irreversible electron transfer process.^39^ On the other hand, the plot of log *I*_PA1_*vs.* log *v* is shown in Fig. 4 part II. As can be seen, log *I*_PA1_ is linearly proportional to log *v* with the slope of 0.5, revealing a pure diffusion-controlled electron transfer process according to Randles–Sevcik equation.^39^ In addition, the plot of *I*_PA1_*vs. v*^1/2^ (Fig. 4 part III) is a straight line, indicating that the process is totally diffusion controlled.^39^

**Fig. 5:**
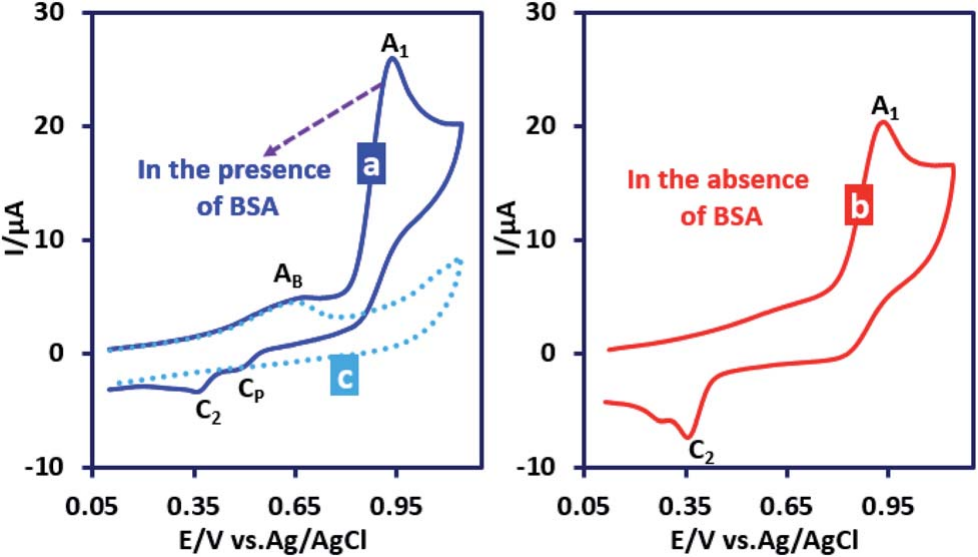
Cyclic voltammograms of PCA (1.0 mM): (a) in the absence, (b) in the presence of BSA (1.0 mM) and (c) cyclic voltammogram of BSA (1.0 mM). Solvent: water (phosphate buffer, pH, 2.0, *c* = 0.2 M)/acetonitrile (50/50 v/v) at GC electrode. Scan rate: 100 mV s^−1^ at room temperature.

In subsequent experiments we investigated the effect of the presence of benzenesulfinic acid (BSA) as a nucleophile on the cyclic voltammograms of PCA. Fig. 5 part I shows the cyclic voltammogram of PCA (1.0 mM) in the presence of BSA (1.0 mM). This voltammogram is compared with the cyclic voltammogram of PCA in the absence of BSA (Fig. 5 part II).

**Scheme 2:**
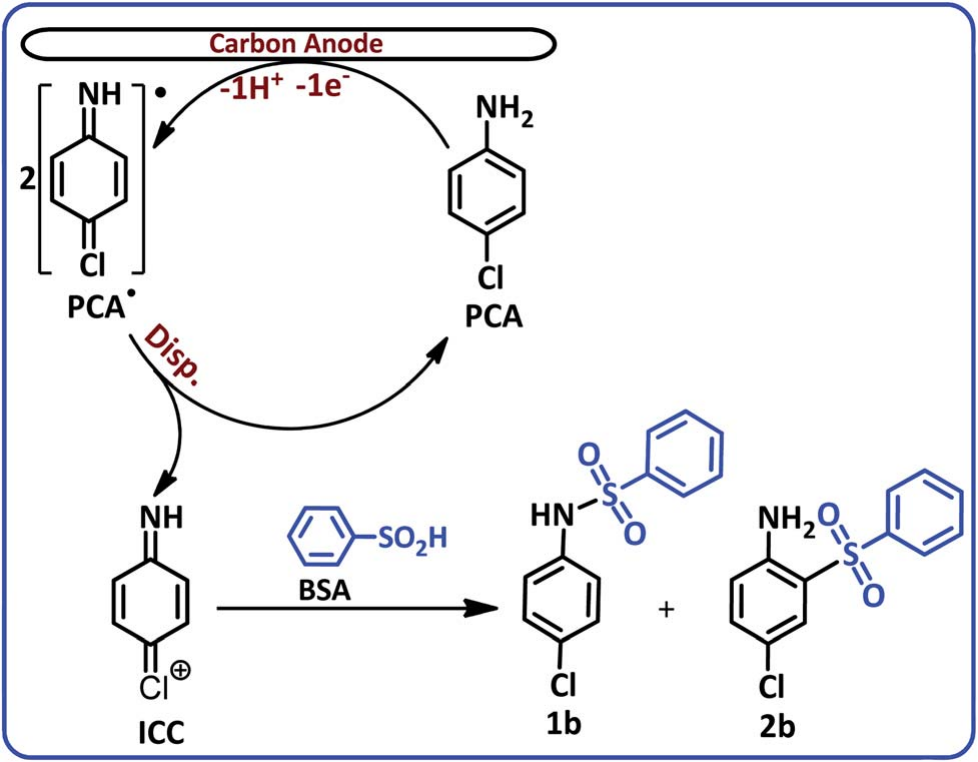
Electrochemical oxidation pathway of PCA in the presence of BSA.

The most noticeable change in the cyclic voltammogram of PCA in the presence of BSA is the decrease in peak C_2_. As described in the previous section, peak C_2_ is due to reduction of PQI to PAP, therefore, a decrease in peak C_2_ current is a sign of a decrease in the concentration of PQI at the electrode surface. In other words, the presence of BSA in the experimental solution has caused electrogenerated ICC consumed in a reaction other than hydrolysis. The general conclusion is that in this condition, ICC reacts with the BSA. Based on these results, along with the spectroscopic data of the isolated products, we propose the following mechanism for the electrochemical oxidation of PCA in the presence of BSA (Scheme 2).

According to this mechanism, PCA at the anode surface is oxidized to its corresponding radical (PCA˙). The disproportionation reaction in the next step, converts PCA˙ into (4-iminocyclohexa-2,5-dien-1-ylidene)chloronium (ICC). The reaction of ICC as an acceptor with nucleophile (BSA) affording *N*-(4-chlorophenyl)benzenesulfonamide (1b) and 4-chloro-2-(phenylsulfonyl)aniline (2b). The infrared spectra of these two compounds clearly show the typical bands of the secondary and primary amines. The FTIR spectrum of 1b, a secondary amine shows only a single band at 3248 cm^−1^, while, the FTIR spectrum of 2b, a primary amine shows two bands at 3483 and 3381 cm^−1^ (see ESI†).

**Scheme 3:**
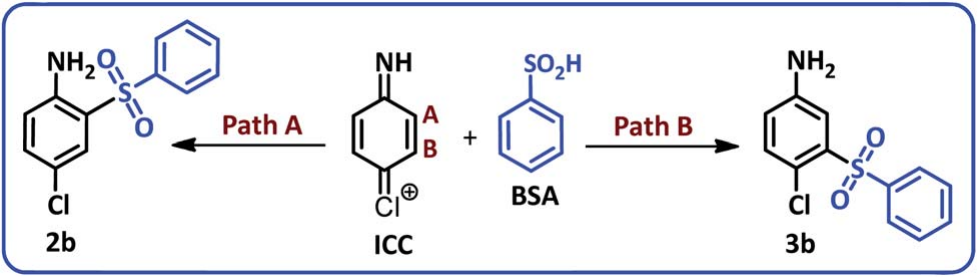
Possible products from reaction of ICC with BSA.

Due to the asymmetry of the ICC, two different paths and in other words two different vacant sites must be considered for nucleophilic addition of BSA to ICC. Consequently, ICC can be attacked by BSA from sites A and B to yield the products 2b and 3b (Scheme 3).

**Fig. 6:**
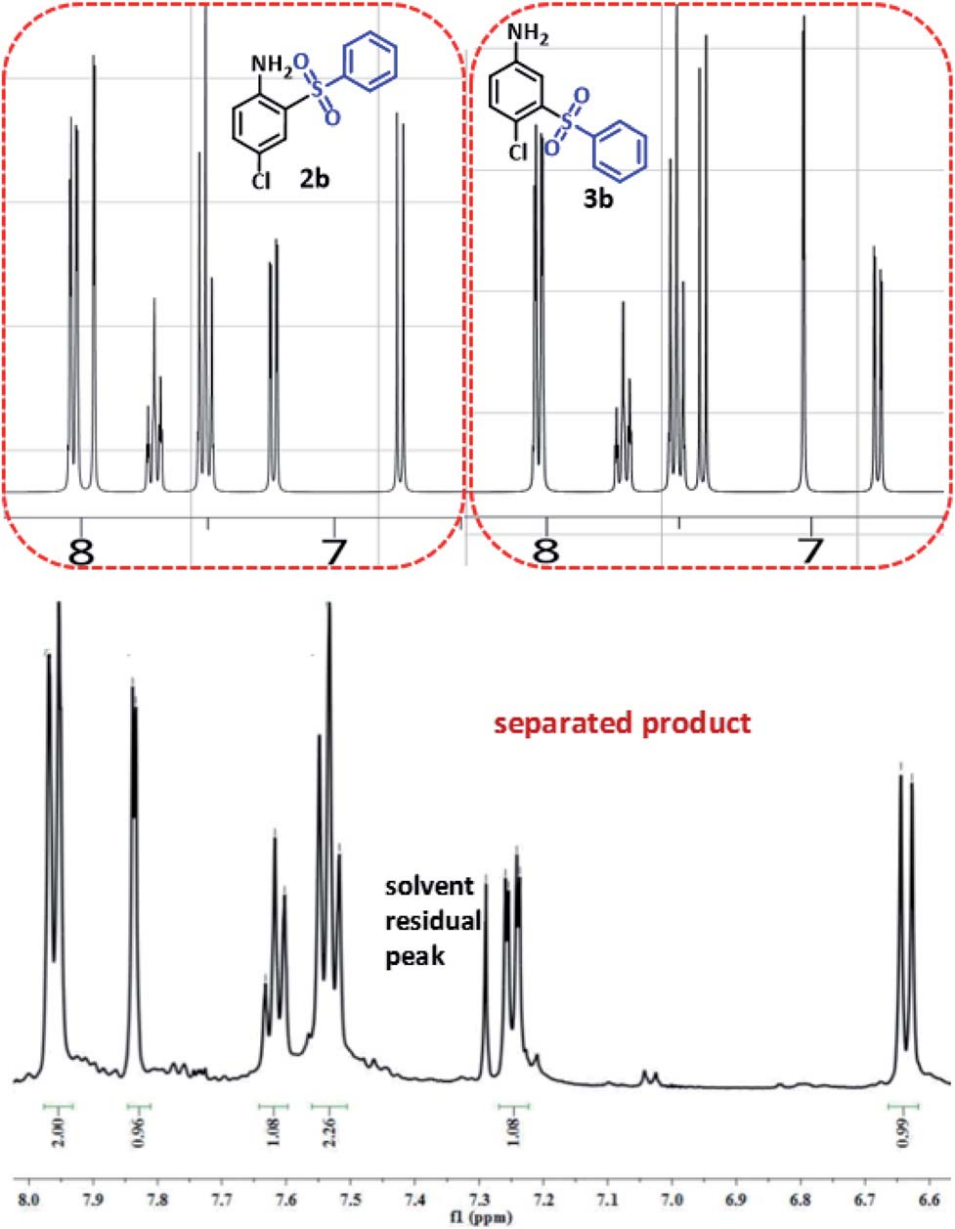
Simulated and experimental ^1^H NMR spectra for compounds 2b, 3b and synthesized diaryl sulfone.

In order to determine the final product, the ^1^H NMR spectra of possible products 2b and 3b were simulated^42^ and compared with ^1^H NMR spectrum of separated product (Fig. 6). This comparison clearly shows that the spectrum of synthesized diaryl sulfone is similar to the simulated spectrum for 2b. This may be due to the formation of intramolecular hydrogen bonding in 2b which can remarkably stabilize it.

**Fig. 7:**
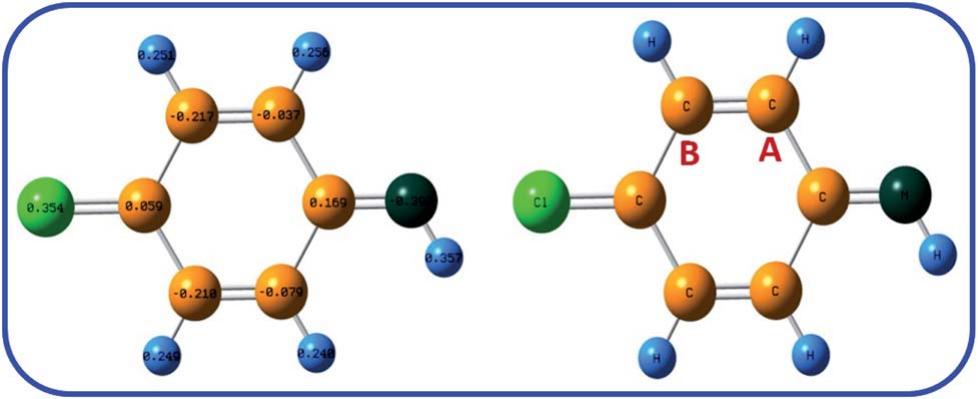
The natural charge of ICC calculated at B3LYP/6-311G levels of theory.

In addition, the Natural Bond Orbital (NBO) analysis was used for calculation of the partial charge in ICC. The B3LYP/6-311G level was used for the calculations. As shown in Fig. 7, the natural charges of carbon-A and carbon-B in ICC are about −0.037 and −0.217 e. This result shows that carbon-A is less negative than carbon-B. The NBO analysis result confirms that the carbon-A is more suitable for chemical reaction than carbon-B.

**Fig. 8:**
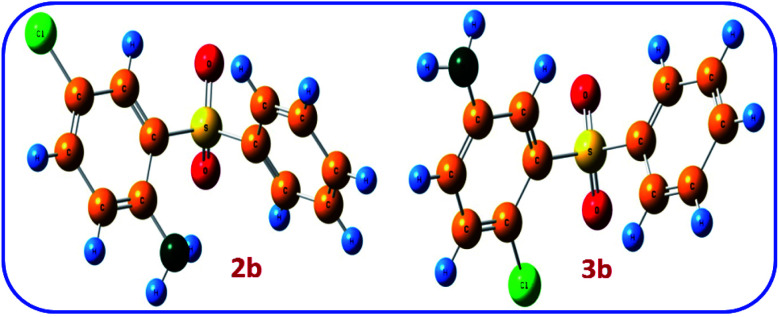
The optimized structures of 2b and 3b at B3LYP/6-311G level of theory.

The thermodynamic stability of two possible products 2b and 3b was also investigated. Fig. 8 shows the optimized structure of 2b and 3b. The comparison of the relative Gibbs free energies of two possible products 2b and 3b, are 25.4 and 0.0 kcal mol^−1^, respectively, which agrees with the results of NBO analysis and confirms the greater stability of 2b.

**Fig. 9:**
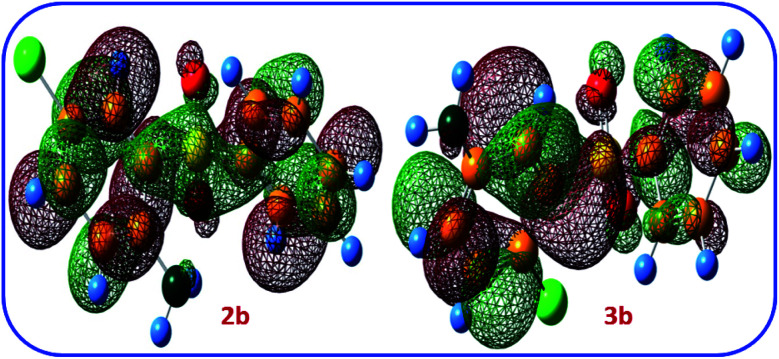
The LUMO orbital structures of possible products.

The LUMO orbital energies of possible products 2b and 3b are also calculated at B3LYP/6-311G level of theory (Fig. 9). The calculated LUMO orbital energies for 2b and 3b are −1.99 and −1.91 eV, respectively. This result show that 2b is more stable than 3b.

**Fig. 10:**
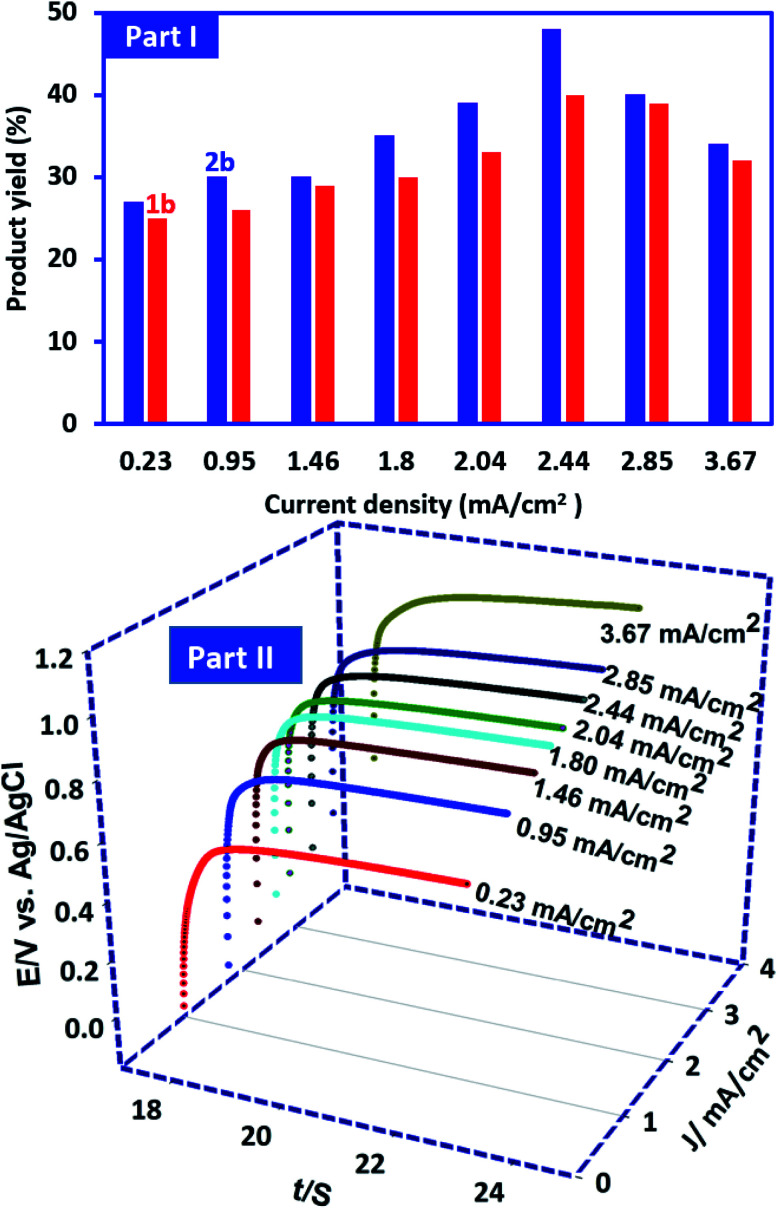
Part I: effect of current density on the yield of 1b and 2b. Charge consumed, 50 C. Part II: the potential–time diagram during constant current electrolysis PCA (0.25 mmol) in the presence of BSA (0.5 mmol) in water (phosphate buffer, pH, 2.0, *c* = 0.2 M)/acetonitrile (50/50 v/v) solution. Electrode rotation rate: 500 rpm.

In this research, in order to facilitate the synthesis of these compounds, a constant current method has also been used. In this method, the current density is one of the most important factors that should be optimized. This factor plays an important role in the purity and the yield of product. In this method, the synthesis of 1b and 2b was performed under the same conditions as described for controlled-potential method. In these experiments the current density varied from 0.23 to 3.67 mA cm^−2^ while the other parameters (temperature = 298 K, PCA amount: 0.25 mmol and BSA amount: 0.5 mmol) are kept constant. It should also be noted that the amount of electricity consumed in these optimizations is kept constant and is equal to the theoretical amount of electricity for 0.25 mmol PCA (50 C). Our results show that the highest yield (48% for 2b and 40% for) 1b was obtained at a current density of 2.44 mA cm^−2^ (Fig. 10, part I). The potential–time diagram during constant current electrolysis is shown in Fig. 10, part II. This figure shows that the potential created by applying the current intensity of 2.44 mA cm^−2^ is about 1 V *vs.* Ag/AgCl. 1 V is a suitable potential applied in controlled-potential method (see Experimental section). The lower product yields at low current intensities may be due to competition between the hydrolysis of ICC (Scheme 1) and the reaction of ICC with BSA (Scheme 2). On the other hand, the higher current densities result in an increase in anodic overpotential and then increase in side reactions such as the oxidation of products and solvent which will lead to a decrease in product yield.

## Conclusions

In this paper, the electrochemical oxidation of 4-chloroaniline as a model compound in aqueous solutions was investigated. Our results show that the 4-chloroaniline is converted to (4-iminocyclohexa-2,5-dien-1-ylidene)chloronium (ICC) after the passage of an electron and a proton and participating in the disproportionation reaction. ICC is an unstable compound and is hydrolyzed to *p*-quinoneimine (PQI) (Scheme 1). Despite this trend, when BSA is added as a nucleophile, the reaction pathway changes due to the higher rate of addition reaction between ICC and BSA (Scheme 2). In this work, we have developed an electrochemical approach for the successful synthesis of potentially important compounds (1b and 2b). The prominent features of this work are, provide new data on the oxidation of 4-chloroaniline in aqueous solutions, developed a new method for the synthesis of potentially important compounds, synthesis at room temperature and atmospheric pressure, using the electrode as an electron source instead of toxic reagents, one-pot synthesis and high energy efficiency.

## Experimental section

### Apparatus and reagents

A SAMA500 potentiostat/galvanostat was used for cyclic voltammetry and controlled-potential coulometry technics. The working and counter electrodes used in the voltammetry experiments were a glassy carbon disc (2.6 mm diameter) and a platinum rod, respectively. The working electrode used in controlled-potential coulometry and macroscale electrolysis was an assembly of four carbon rods (31 cm^2^), while a large stainless steel plate constituted the counter electrode. The working electrode potentials were measured *vs.* Ag/AgCl (all electrodes from AZAR electrodes). Benzenesulfinic acid (sodium salt), *p*-chloroaniline, acetic acid, perchloric acid and phosphoric acid were obtained from commercial sources.

### General procedure for synthesis of 1b, 2b

In an undivided cell equipped with four carbon rods as anode and a large stainless steel plate as cathode, a mixture of water (phosphate buffer, pH, 2.0, *c* = 0.1 M)/acetonitrile (50/50 v/v) (*ca.* 80 mL) containing 4-chloroaniline (1.0 mM) and benzenesulfinic acid (sodium salt) (2 mM) was electrolyzed at 1 V *vs.* Ag/AgCl. The electrolysis was terminated when the current decayed to 5% of its original value. At the end of the electrolysis, the cell was placed overnight. The precipitated light brown was collected by filtration. The purification and separation of two compounds 1b and 2b was performed by thin layer chromatography (*n*-hexane/ethyl acetate 8/1).

#### *N*-(4-Chorophenyl)benzenesulfonamide (1b)

Light brown; isolated yield 43%, mp = 106–108 °C; ^1^H NMR: *δ* ppm (500 MHz, CDCl_3_): 7.07 (d, *J* = 10 Hz, 2H, aromatic), 7.21 (d, *J* = 10 Hz, 2H, aromatic), 7.40 (broad-s, 1H, N–H), 7.48 (t, 2H aromatic), 7.58 (t, 1H, aromatic), 7.81 (d, *J* = 10 Hz, 2H, aromatic); ^13^C NMR: *δ* ppm (125 MHz, CDCl_3_): 123.0, 127.2, 129.3, 129.4, 131.0, 133.3, 134.9, 138.6; IR (KBr) (cm^−1^): 3248 (medium, N–H), 1490 (medium C=C), 1331 and 1160 (strong, S=O), 1090, 911, 685, 631, 584; MS (*m*/*z*) (EI, 70 eV) (relative intensity): 77 (100), 126 (95), 99 (70), 51 (68), 267 (M^+^, 77).

#### 4-Chloro-2-(phenylsulfonyl)aniline (2b)

Dark brown, isolated yield 52%, mp = 90–93 °C; ^1^H NMR: *δ* ppm (500 MHz, CDCl_3_): 4.99 (broad, 2H, NH_2_), 6.63 (d, *J* = 8.5 Hz, 1H, aromatic), 7.25 (dd, *J* = 8.5 and 2.8 Hz, 1H, aromatic), 7.53 (t, 2H, aromatic), 7.62 (t, 1H, aromatic), 7.84 (d, *J* = 2.8, 1H, aromatic), 7.96 (d, *J* = 8.6, 2H, aromatic); ^13^C NMR: *δ* ppm (125 MHz, CDCl_3_): 119.3, 122.6, 127.0, 129.1, 129.2, 133.5, 135.0, 141.1, 144.5; IR (KBr) (cm^−1^): 3483–3381 (medium, NH_2_), 1446 (medium C=C), 1297 and 1145 (strong, S=O), 1067, 688, 743, 599; MS (*m*/*z*) (EI, 70 eV) (relative intensity): 267 (M^+^, 100), 167 (85), 202 (80), 149 (75), 51 (70).

## Conflicts of interest

The authors declare no conflict of interest.

## Supplementary Material

RA-010-D0RA05680D-s001
