# Supplementary material for: Electrochemical study of 4-chloroaniline in a water/acetonitrile mixture. A new method for the synthesis of 4-chloro-2-(phenylsulfonyl)aniline and N-(4-chlorophenyl)benzenesulfonamide
Source: RSC Adv. 2020 Aug 26;10(52):31563–9. doi: 10.1039/d0ra05680d (PMC9056402; doi:10.1039/d0ra05680d)
Supplement: RA-010-D0RA05680D-s001 [file RA-010-D0RA05680D-s001.pdf]

Electronic Supplementary Material (ESI) for RSC Advances.
This journal is © The Royal Society of Chemistry 2020

Electrochemical Study of 4-Chloroaniline in Water/Acetonitrile Mixture. A new Method for the Synthesis of 4-Chloro-2-(phenylsulfonyl)aniline and *N*-(4-Chlorophenyl)benzenesulfonamide

Niloofar Mohamadighader,[a] Mahnaz Saraei,[a] Davood Nematollahi,[*b] Hamed Goljani[b]

[a]Department of Chemistry, Payame Noor University, Tehran, Iran

[b]Faculty of Chemistry, Bu-Ali-Sina University, Hamedan 65174, Iran.

nemat@basu.ac.ir.

No		Page
1	FT-IR spectrum of **1b**……………………………………………………..	2
2	^{1}H NMR spectrum of **1b**………………………………………………..	3
3	^{1}H NMR spectrum of **1b** (Expanded)…………………………………..	4
4	^{13}C NMR spectrum of **1b**……………………………………………….	5
5	^{13}C NMR spectrum of **1b** (Expanded)…………………………………..	6
6	MS spectrum of **1b**……………………………………………………..	7
7	FT-IR spectrum of **2b**……………………………………………………..	8
8	^{1}H NMR spectrum of **2b**..	9
9	^{1}H NMR spectrum of **2b** (Expanded)…………………………………..	10
10	^{13}C NMR spectrum of **2b**……………………………………………….	11
11	^{13}C NMR spectrum of **2b** (Expanded)…………………………………..	12
12	Ms spectrum of **2b**……………………………………………………...	13
13	Cyclic voltammograms of **PCA** at different concentrations……………..	14

FT-IR spectrum of 1b

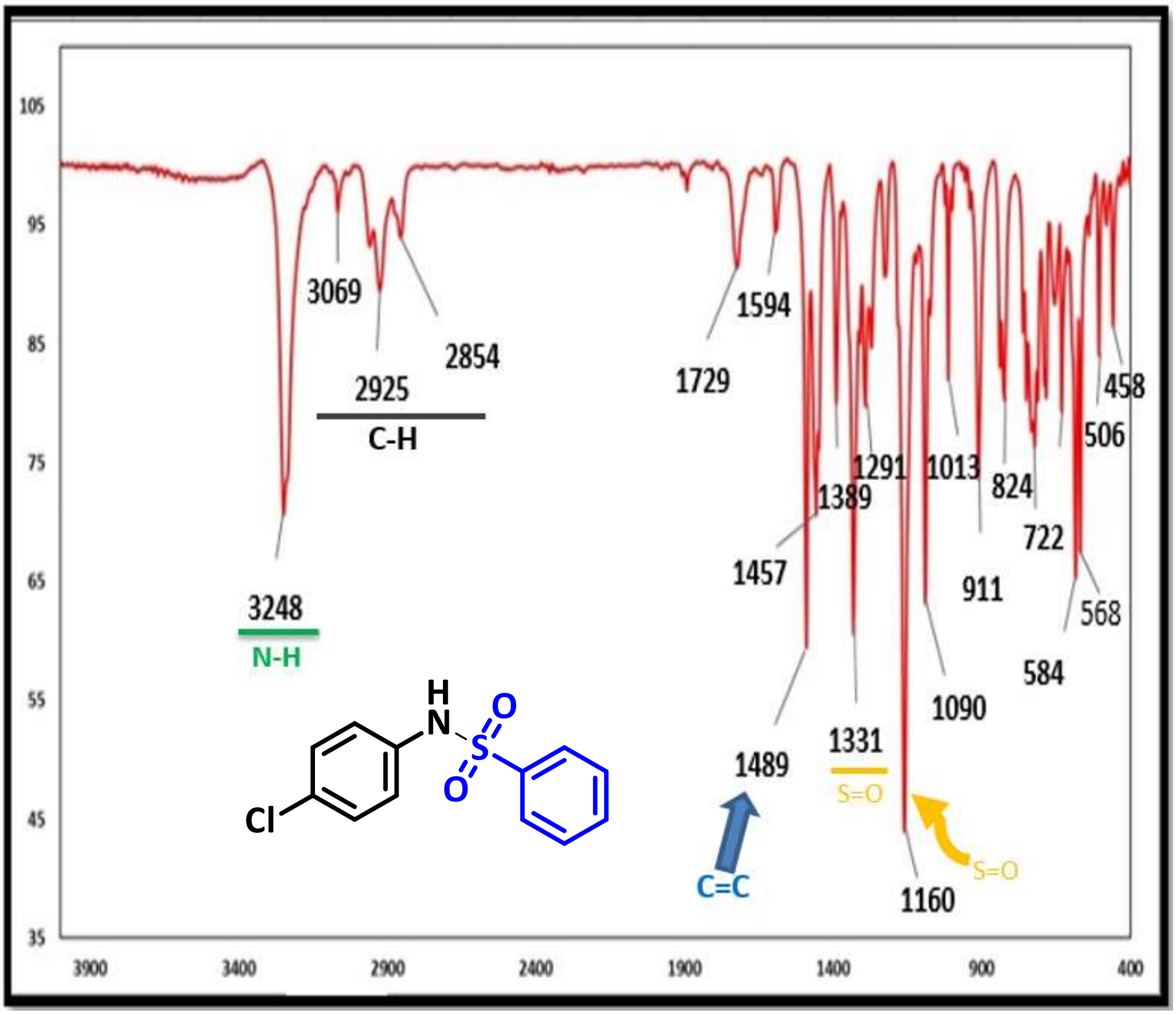

^{1}H NMR spectrum of 1b

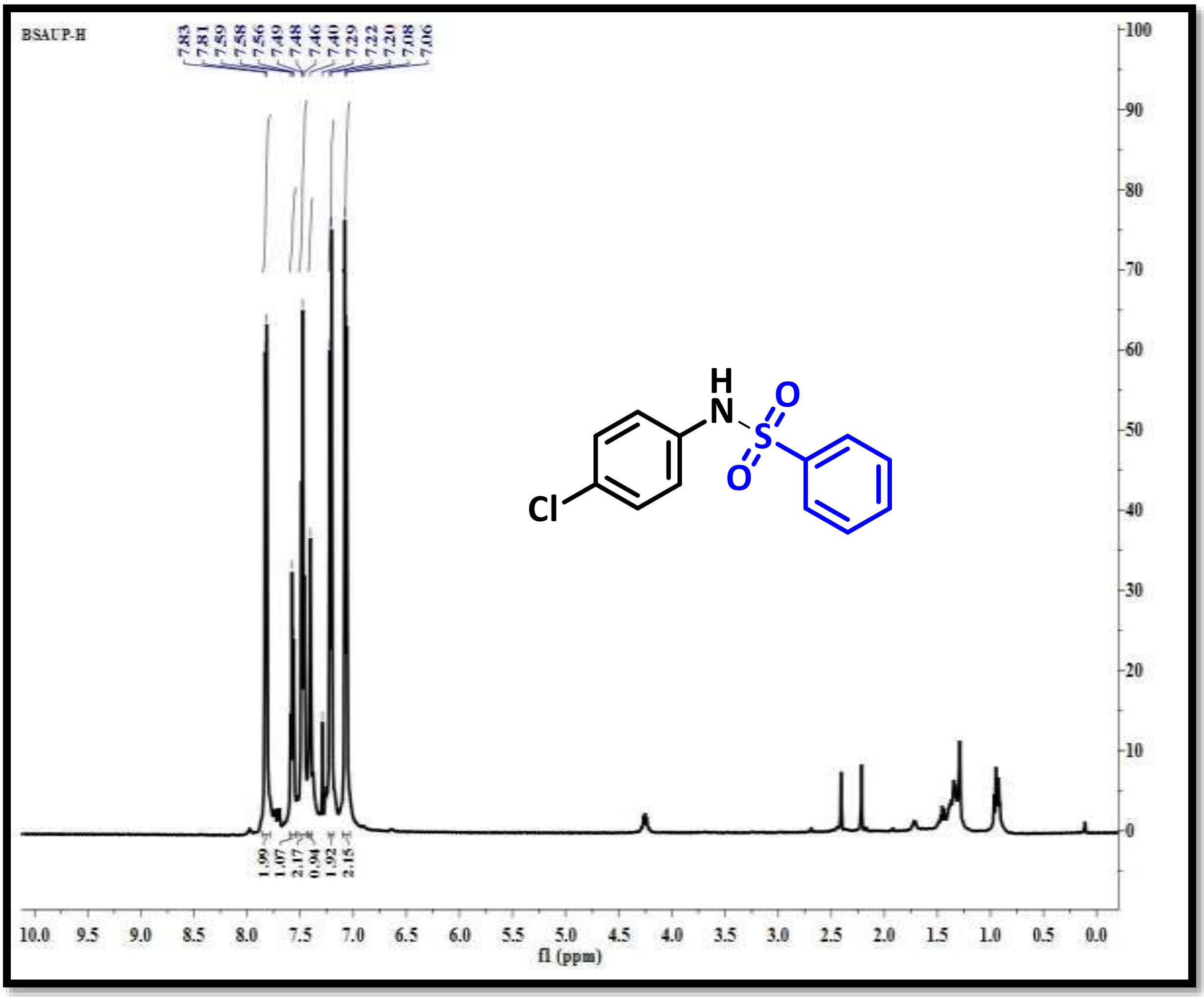

Expanded ^{1}H NMR spectrum of 1b

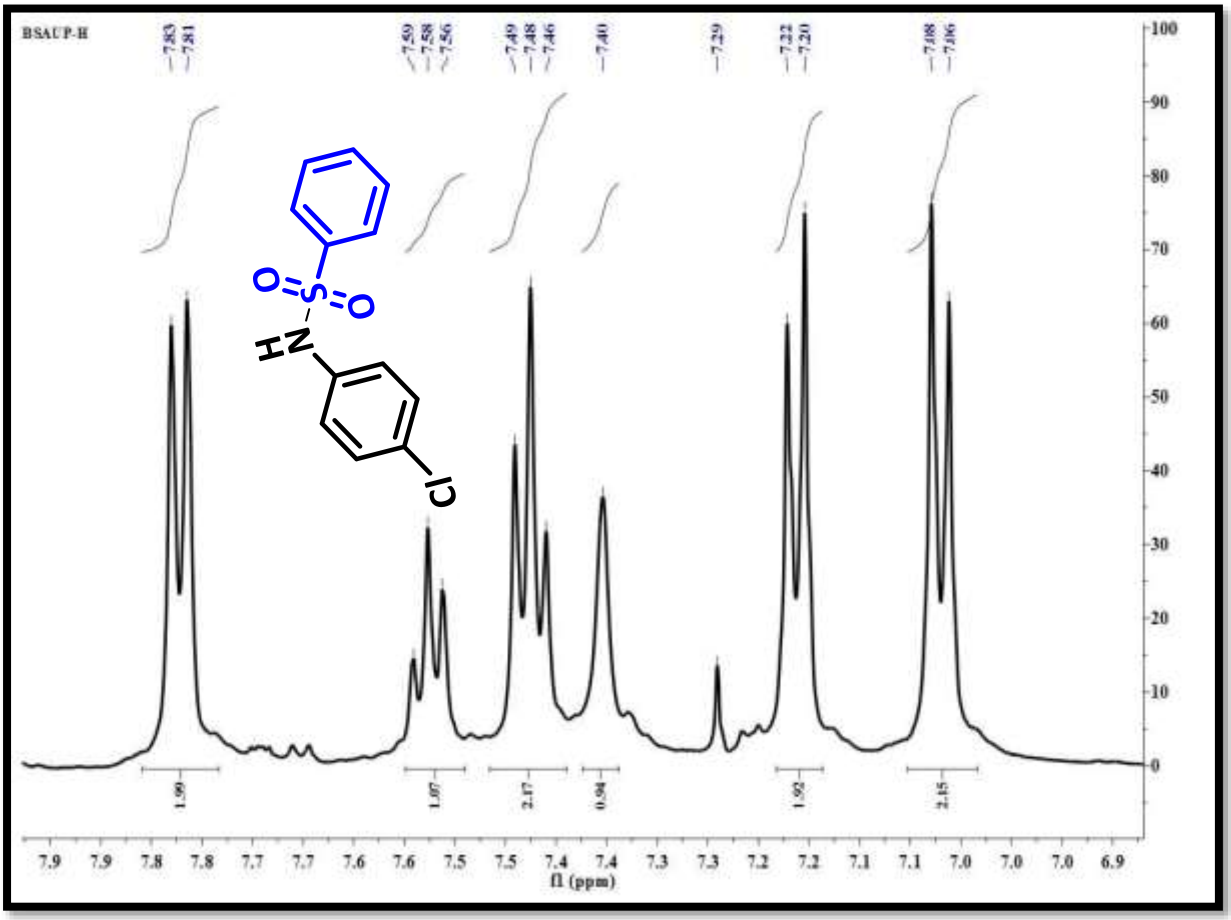

^{13}C NMR spectrum of 1b

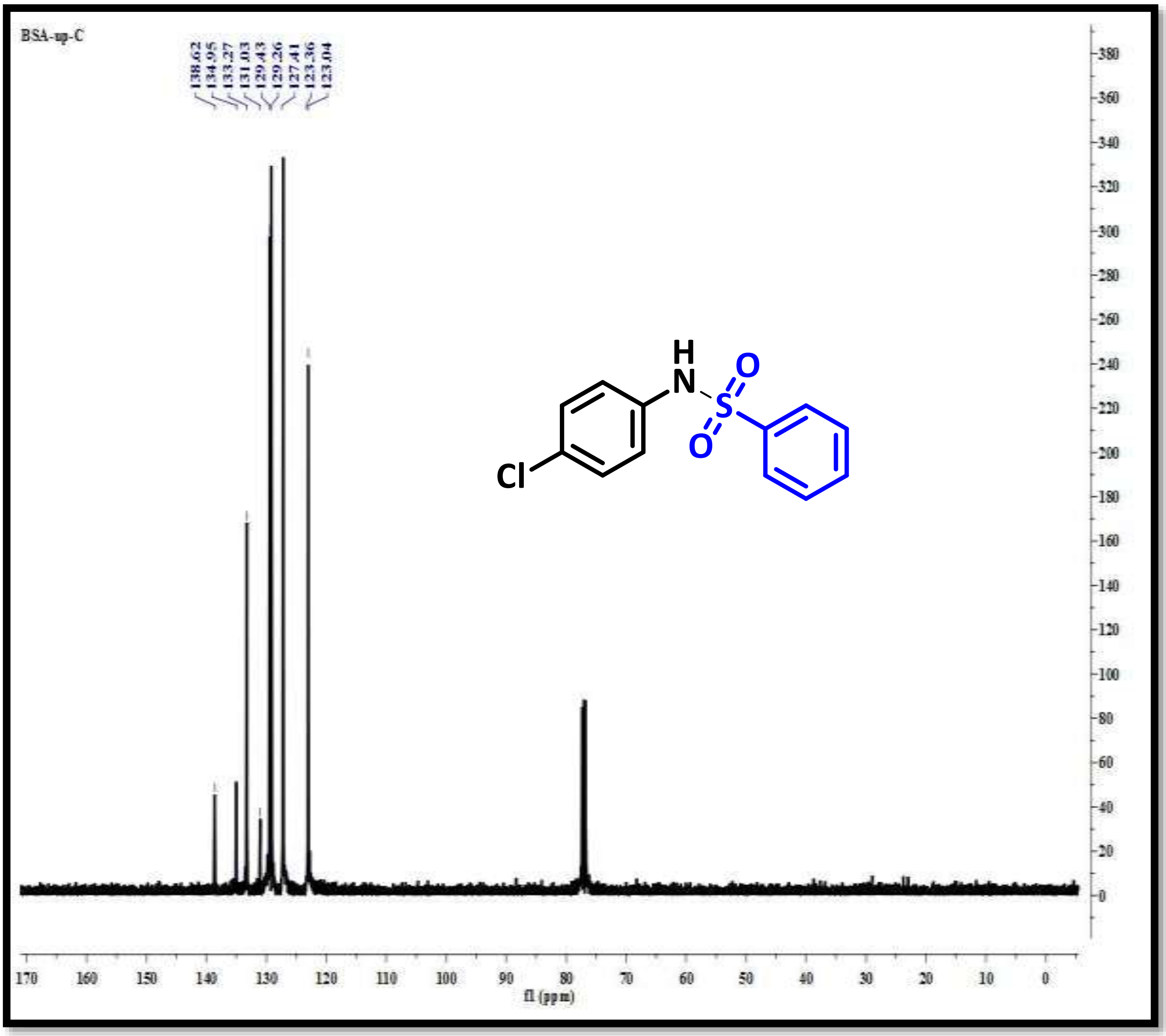

Expanded ^{13}C NMR spectrum of 1b

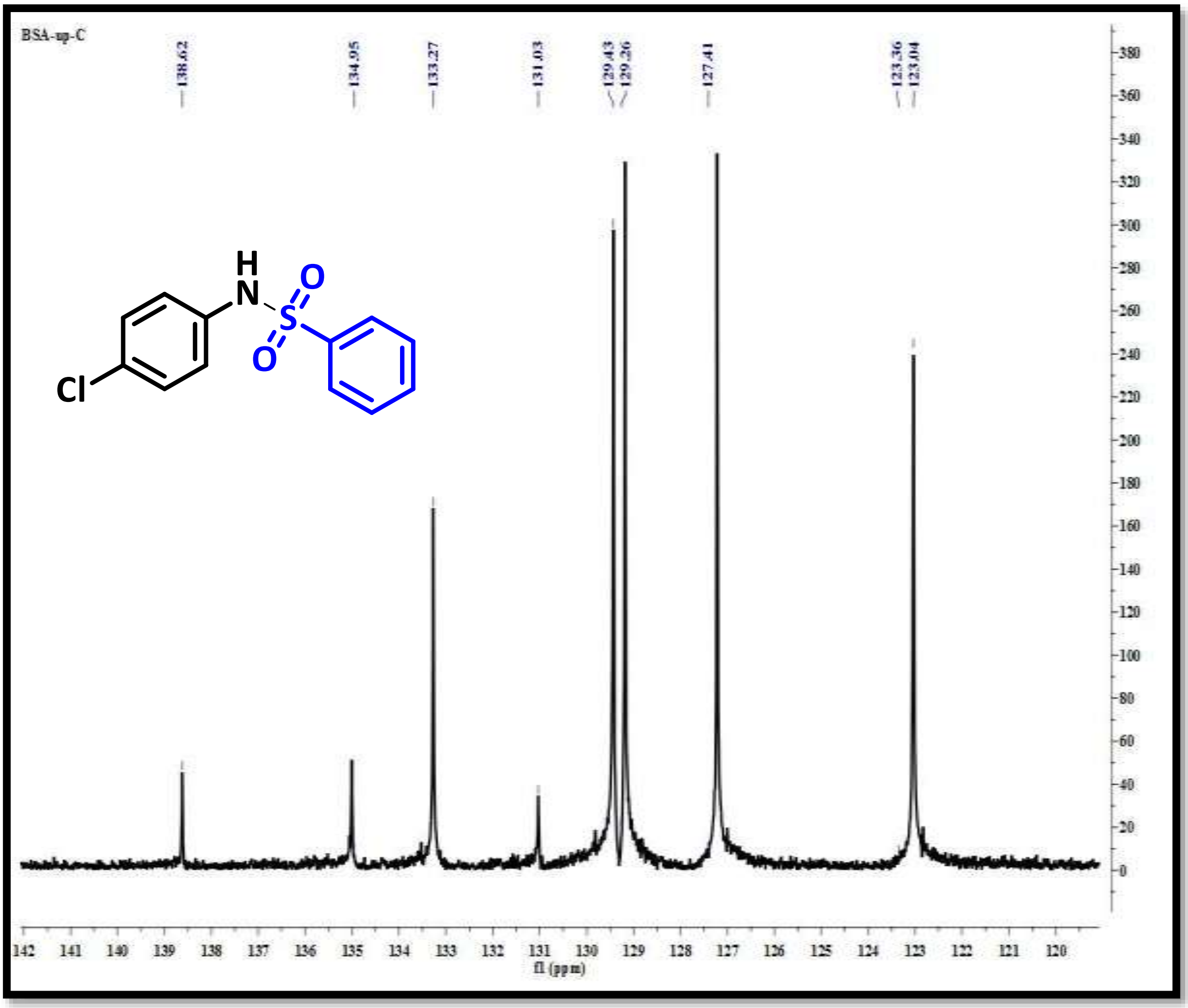

MS spectrum of 1b

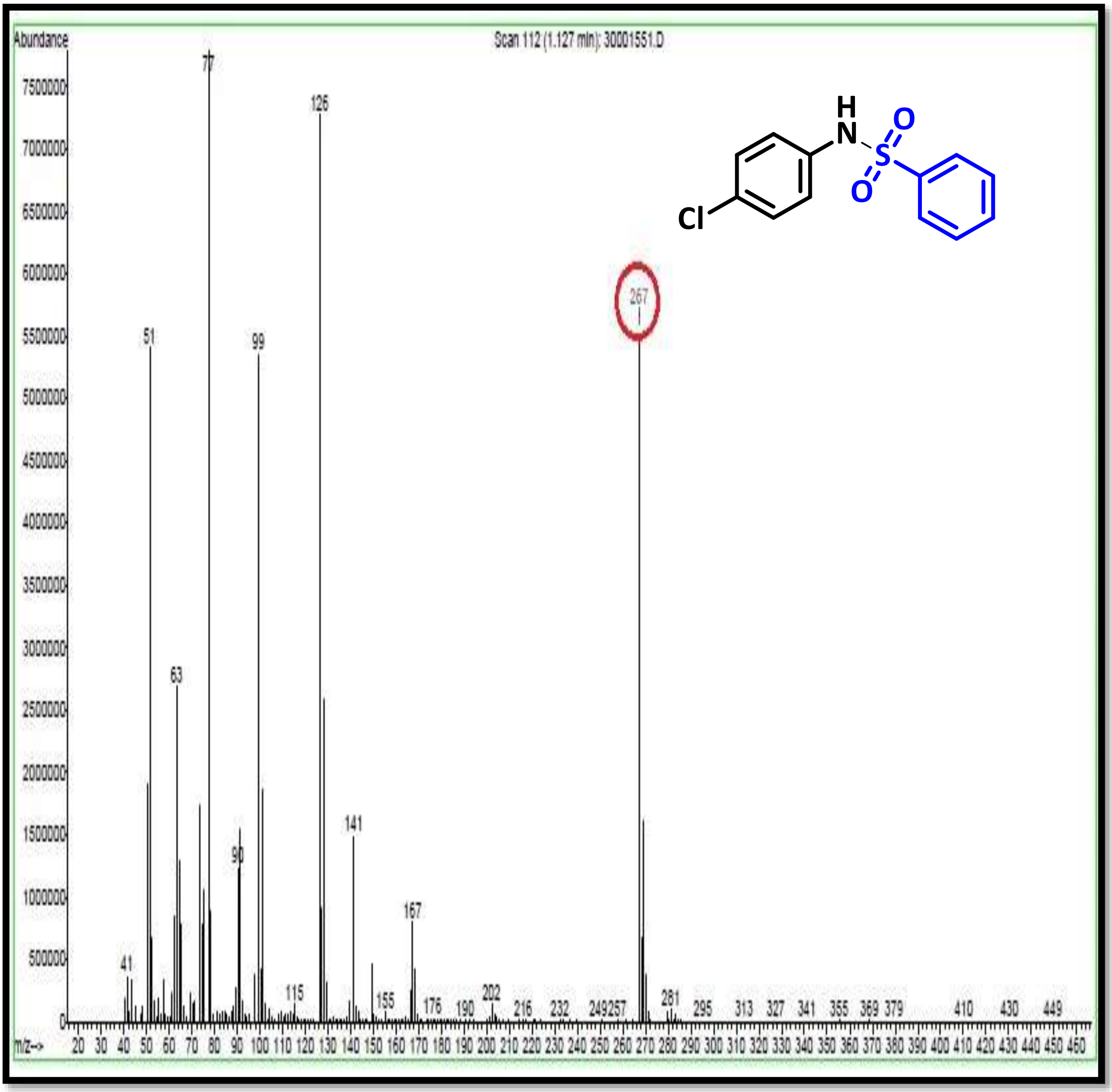

FT-IR spectrum of 2b

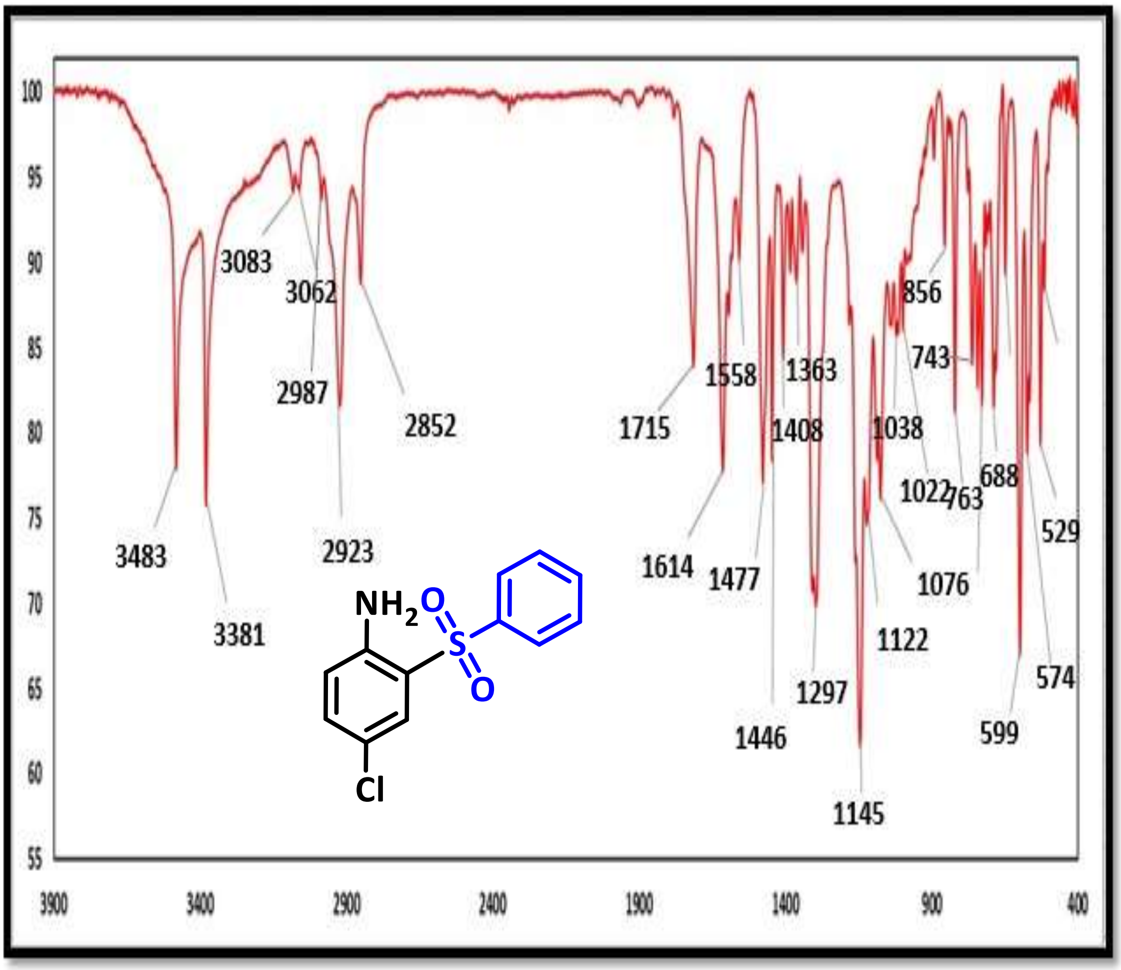

^{1}H NMR spectrum of 2b

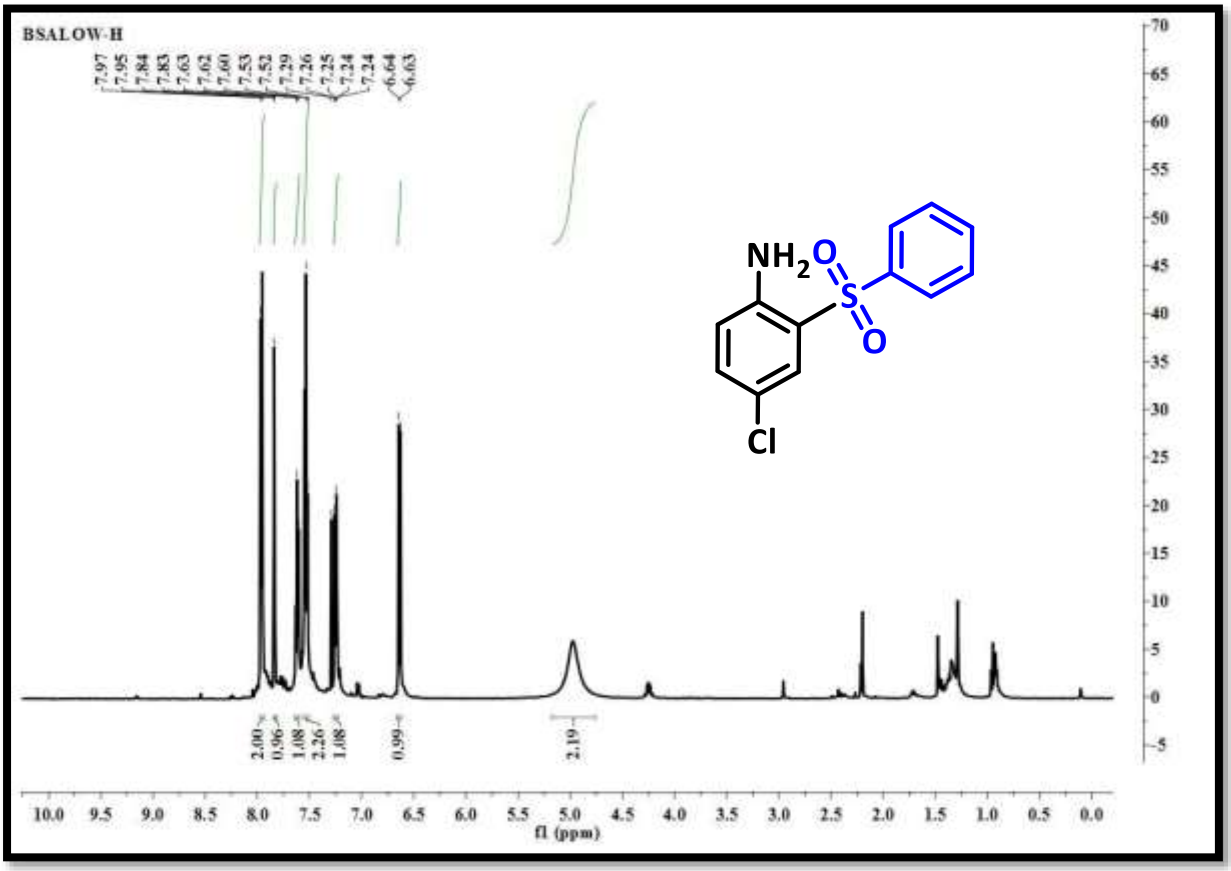

Expanded ^{1}H NMR spectrum of 2b

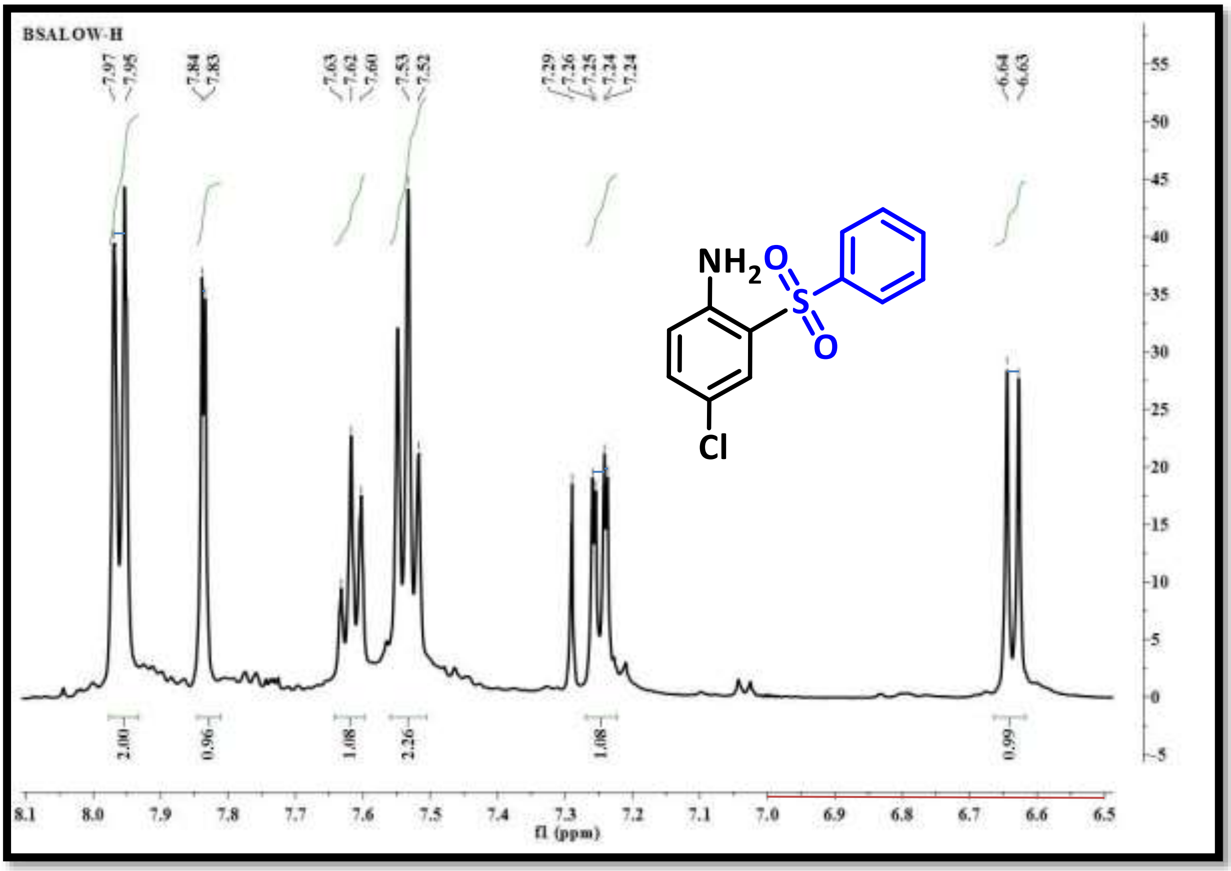

¹³C NMR spectrum of 2b

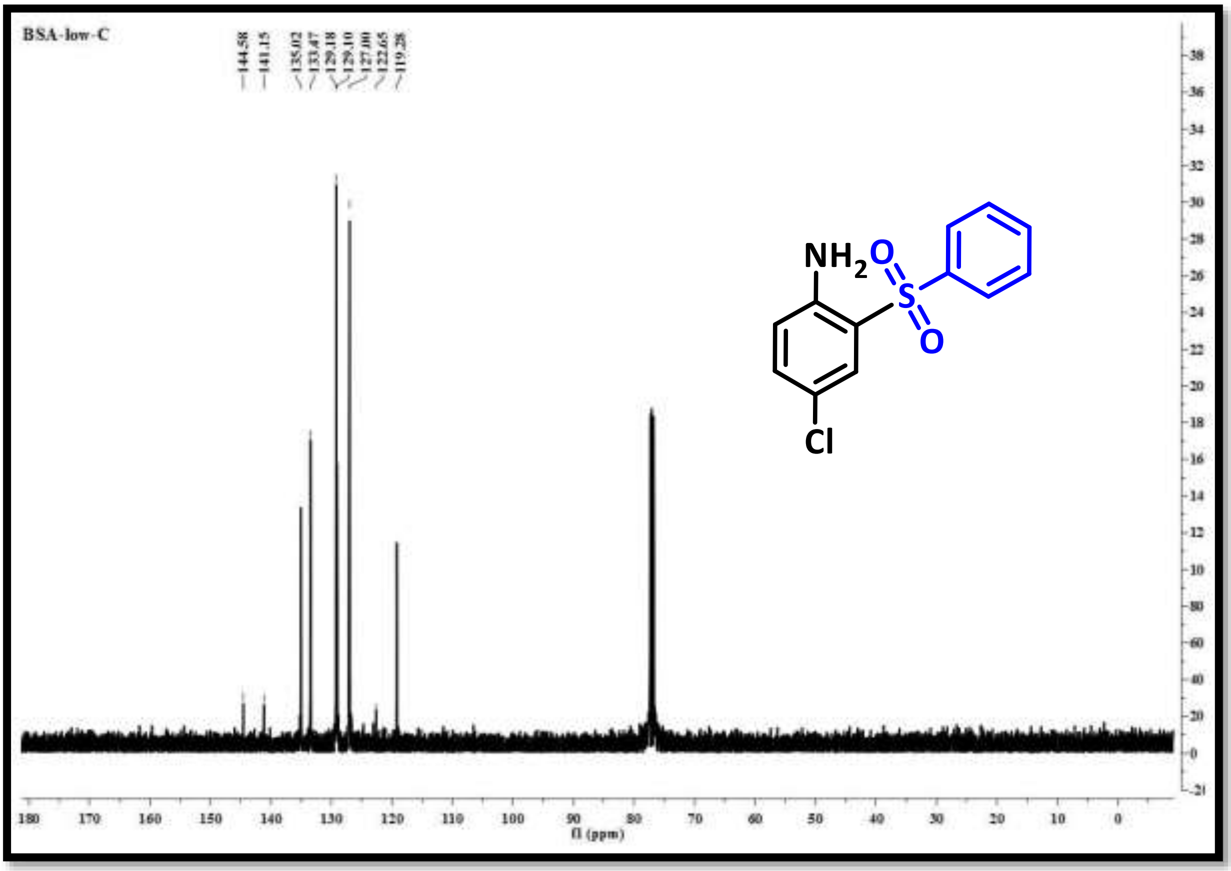

Expanded ^{13}C NMR of 2b

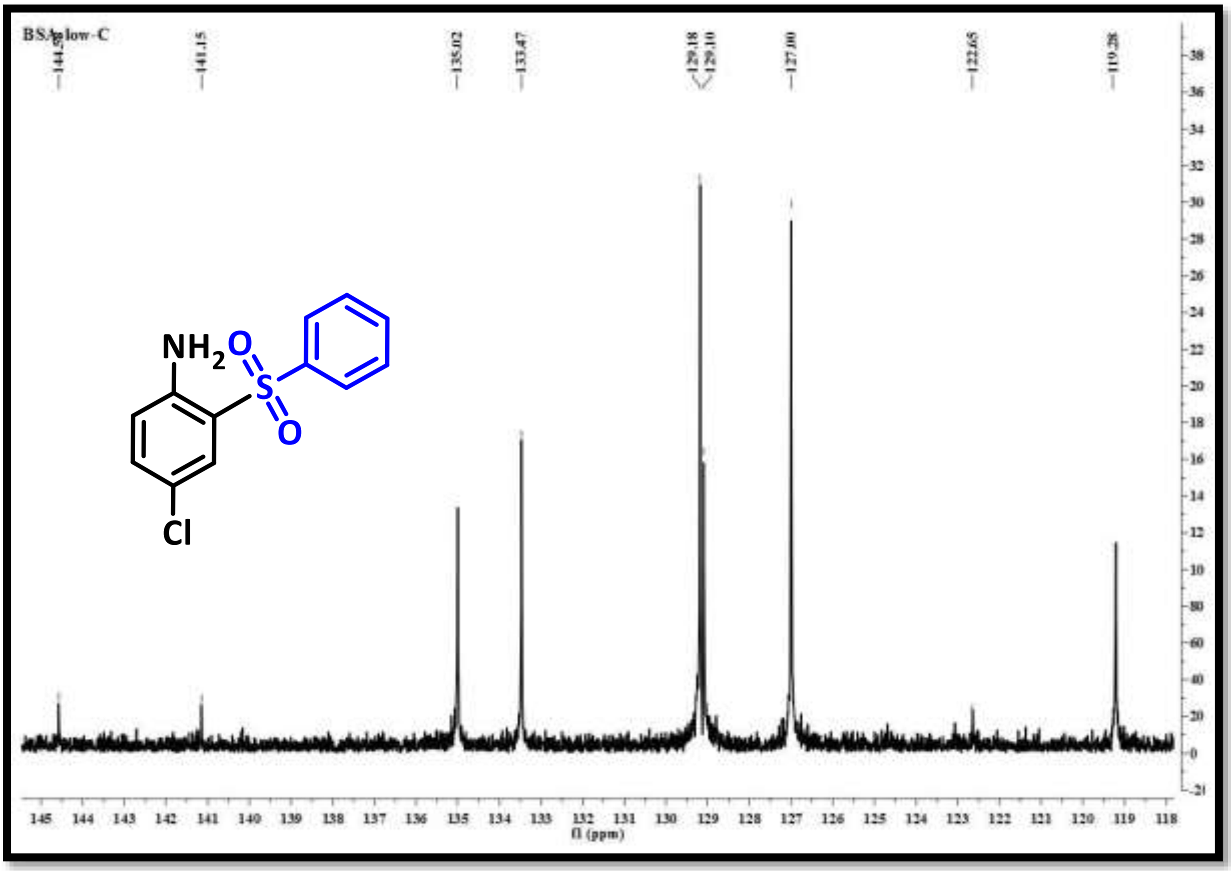

Ms spectrum of 2b

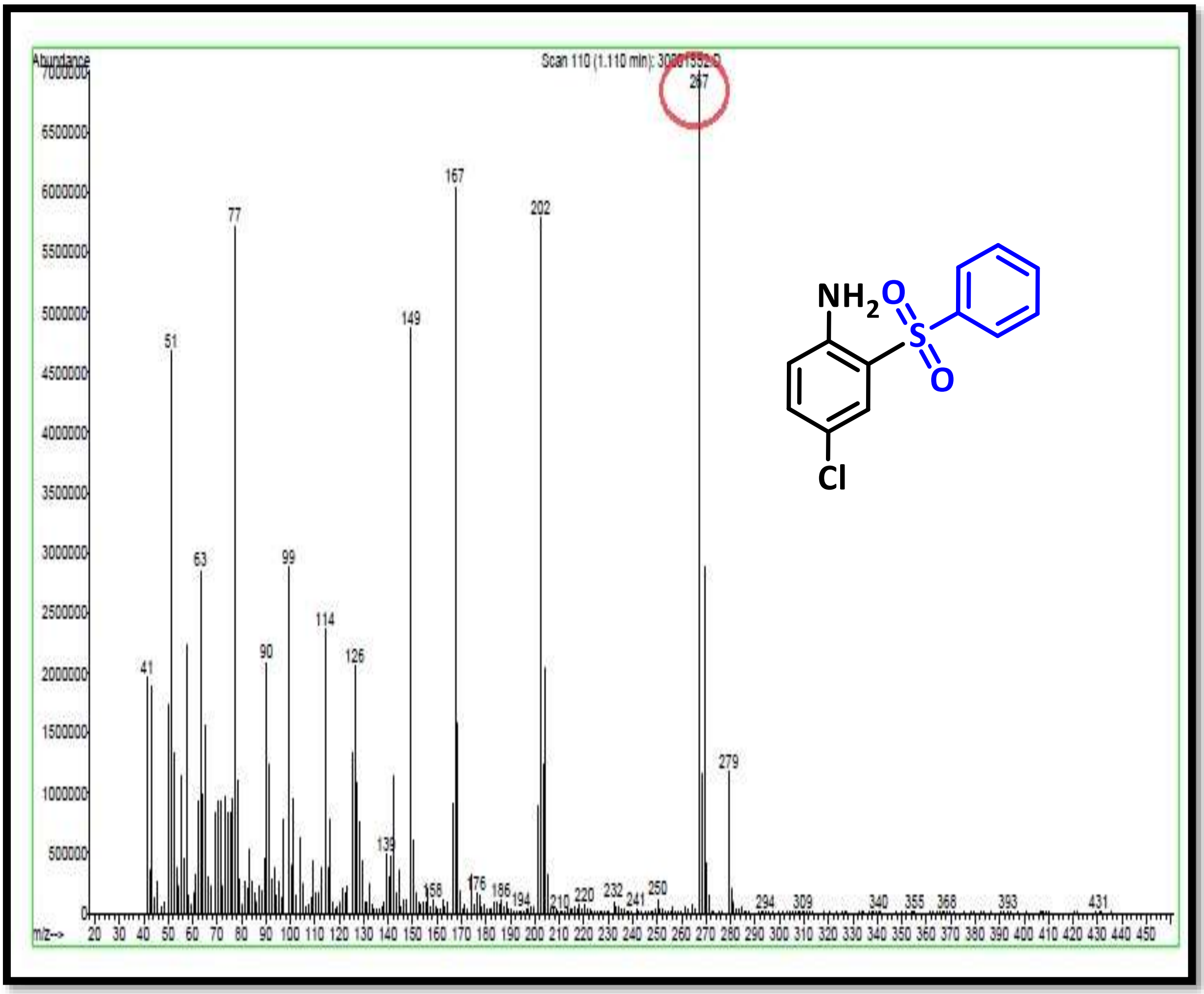

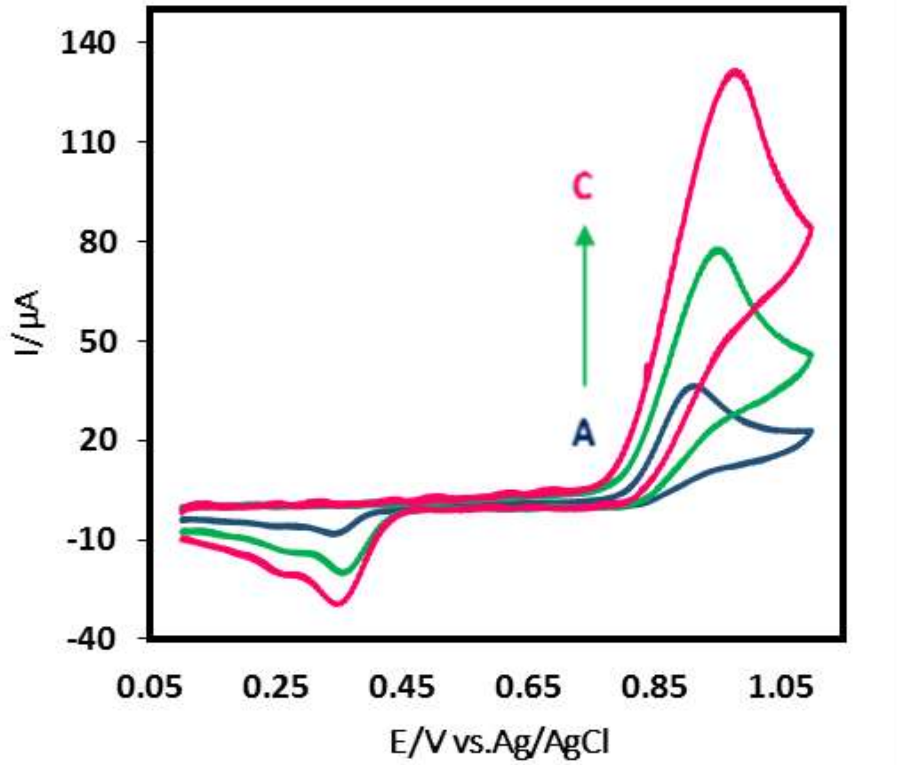

Figure S1: Cyclic voltammograms of **PCA** at different concentrations (A: 1, B: 3 and C: 6 mM) at 100 mV/s. Solvent: Water (0.1 M perchloric acid)/acetonitryl (50/50, v/v) mixture. Working electrode: Glassy carbon electrode. Temperature: room temperature.
